# CONECT-6: a case-finding tool to identify patients with complex health needs

**DOI:** 10.1186/s12913-021-06154-4

**Published:** 2021-02-17

**Authors:** Catherine Hudon, Mathieu Bisson, Marie-France Dubois, Yohann Chiu, Maud-Christine Chouinard, Nicole Dubuc, Nicolas Elazhary, Véronique Sabourin, Alain Vanasse

**Affiliations:** 1 Department of Family Medicine and Emergency Medicine, University of Sherbrooke, 3001 12e Avenue N, Sherbrooke, QC J1H 5H3 Canada; 2 Department of Family Medicine and Emergency Medicine, University of Sherbrooke, Pavillon Z7-Room 3007, 3001, 12e Avenue Nord, Sherbrooke, QC J1H 5N4 Canada; 3 Nursing Faculty, University of Montreal, Pavillon Marguerite-d’Youville, C.P. 6128 succ. Centre-ville, Montréal, QC H3C 3J7 Canada; 4 Integrated University Health and Social Services Centre of Saguenay–Lac-Saint-Jean, 930 rue Jacques-Cartier E, Chicoutimi, QC G7H 7K9 Canada

**Keywords:** Case-finding tool, Complexity, Chronic diseases, Ambulatory care sensitive condition, Case management, Frequent user

## Abstract

**Background:**

Early identification of patients with chronic conditions and complex health needs in emergency departments (ED) would enable the provision of services better suited to their needs, such as case management. A case-finding tool would ultimately support ED teams to this end and could reduce the cost of services due to avoidable ED visits and hospitalizations. The aim of this study was to develop and validate a short self-administered case-finding tool in EDs to identify patients with chronic conditions and complex health needs in an adult population.

**Methods:**

This prospective development and initial validation study of a case-finding tool was conducted in four EDs in the province of Quebec (Canada). Adult patients with chronic conditions were approached at their third or more visit to the ED within 12 months to complete a self-administered questionnaire, which included socio-demographics, a comorbidity index, the reference standard INTERMED self-assessment, and 12 questions to develop the case-finding tool. Significant variables in bivariate analysis were included in a multivariate logistic regression analysis and a backward elimination procedure was applied. A receiver operating characteristic (ROC) curve was developed to identify the most appropriate threshold score to identify patients with complex health needs.

**Results:**

Two hundred ninety patients participated in the study. The multivariate analysis yielded a six-question tool, COmplex NEeds Case-finding Tool – 6 (CONECT-6), which evaluates the following variables: low perceived health; limitations due to pain; unmet needs; high self-perceived complexity; low income; and poor social support. With a threshold of two or more positive answers, the sensitivity was 90% and specificity 66%. The positive and negative predictive values were 49 and 75% respectively.

**Conclusions:**

The case-finding process is the essential characteristic of case management effectiveness. This study presents the first case-finding tool to identify adult patients with chronic conditions and complex health needs in ED.

**Supplementary Information:**

The online version contains supplementary material available at 10.1186/s12913-021-06154-4.

## Background

Frequent users of emergency departments (ED) represent a small proportion of patients (approximately 5%) who account for 30 to 50% of all ED visits [1–3]. Frequent use of ED is often defined as three or four visits or more in the last year [4–11]. Fragmented, episodic and poorly coordinated, the care that these patients receive through the ED is often suboptimal in relation to their needs [12–14]. In many cases, this significant use of ED could be avoided by providing adequate care upstream [4, 15]. Over 80% of frequent users of ED present chronic conditions for which adequate ambulatory care can prevent deterioration or complications requiring visits to the ED or hospitalizations [16], and a majority present several conditions simultaneously [17]. According to the Canadian Institute for Health Information [18], these conditions, called ambulatory care sensitive conditions (ACSC), include angina, asthma, chronic obstructive pulmonary disease, diabetes, grand mal status and other epileptic convulsions, heart failure and pulmonary edema, and hypertension. Even though adequate care can prevent complications, a large proportion of hospital activities are devoted to ED visits and hospitalizations linked to these conditions [19].

### Importance

For some frequent ED users with ACSC, the simultaneous presence of psychological and/or social issues can create a complexity that ends up interfering with usual care [20–23]. The Agency for Healthcare Research and Quality Multiple Chronic Conditions Research Network conceptualizes complexity as the gap between an individual’s needs and the ability of health services to meet those needs [24]. The greater the complexity, i.e. the greater the gap between an individual’s needs and the ability of services increases, the greater the challenge in adjusting care to bridge this gap. These patients may attempt unsuccessfully to fulfill their unmet health needs by using care and services, such as the ED, generating considerable costs, and present poorer health indicators including high mortality rates [25]. Early identification of frequent ED users with complex health needs using a case-finding tool could enable intervening upstream and offering services better suited to their needs. Case management, for example, is increasingly recognized internationally as an appropriate intervention, in complex situations, to improve services and the healthcare system’s capacity to satisfy the particular needs of some patients [1, 26–30].

### Goals of this investigation

While a few clinical tools are available in ED to identify certain at-risk patients, such as older adults at risk of losing their autonomy or exhibiting frailty (e.g. Program on Research for Integrating Services for the Maintenance of Autonomy - PRISMA 7, Identification of Seniors at Risk - ISAR) [31, 32] none enable the specific identification of patients with complex health needs. A recent scoping review to find a short (less than 15 min) and valid screening tool for identifying all adults with complex health needs at risk of high use of healthcare services concluded that most tools targeted older adults [33]. The only questionnaire available for an adult population was the INTERMED Self-Assessment – IMSA [34]. However, despite its validity and interest in terms of complexity measure, administration length and score calculation are too long for a case finding purpose in EDs [33]. The aim of this study was to develop and validate a case-finding tool to identify patients with complex health needs, in an adult population with chronic conditions. Our purpose was to develop a rapid (less than 2 min), self-administered 6–8-item (yes or no answers) case-finding tool.

## Methods

### Study design and settings

This was a multi-centre prospective development and initial validation study of a self-administered questionnaire. The study was approved by the Ethics Review Board of the *Estrie Integrated University Health and Social Services Centre (CIUSSS de l’Estrie) - Sherbrooke University Hospital Centre.*

### Selection of participants

The study was conducted in four EDs affiliated with the Estrie Integrated University Health and Social Services Centre (IUHSSC) and the Saguenay-Lac-Saint-Jean IUHSSC, in two Quebec (Canada) regions. The four EDs are located in three urban areas [35], with a population of respectively 26,669, 145,949 and 161,323 inhabitants in 2016 [36]. Participants were identified at their third or more visit to the ED within 12 months. This cut-off was chosen to increase prevalence of complexity in the sample and avoid screening patients with low risk of complexity. They were approached to participate if they were adults (≥ 18 years), had three or more visits to the ED within 12 months and presented at least one ACSC (angina, asthma, chronic obstructive pulmonary disease, diabetes, grand mal status and other epileptic convulsions, heart failure and pulmonary edema, or hypertension). They were excluded if they had a critical situation requiring urgent care or if they had already participated in this study because of a previous ED visit during the data collection period.

### Interventions

Four research assistants (registered nurses) were present in the EDs (one per ED) 35 h a week, at different moments of the day or evening, for consecutive sampling [37] between January and April 2019. They had access to the electronic registries of the ED to identify eligible participants, including their ACSCs diagnosis. They received 3 h of training on the study and completion of the questionnaire from the research team. For every person approached meeting the inclusion criteria (electronic medical record), research assistants had to complete a recruitment sheet to collect information about how many people completed the questionnaire, how many had to leave or how many refused to participate and for which reason.

After explaining the study and obtaining informed consent, the patient was invited by the research assistant to complete the self-administered questionnaire, in French or in English. The participant did this in a quiet room while they were waiting or at a more appropriate moment later on to avoid interfering with ED care. For participants with low literacy, the research assistant could assist them with the questionnaire.

### Measurements

The questionnaire included socio-demographics (age, sex, native language, occupation, marital status, income), the Disease Burden Morbidity Assessment (DBMA) [38, 39] (comorbidity score), the reference standard IMSA [34, 35], and 12 preliminary questions to develop the case-finding tool, which are available in Additional file 1. Data collection took approximately 40 min per patient (time to explain the study, obtain consent, complete the questionnaire).

#### Questions included in the development of the case-finding tool

We selected 12 questions, already validated, that are associated with complex health needs, based on previous work [40–43]. These questions concerned perceived healthcare status (Statistics Canada) [44], insurance healthcare plan [45], social support (Statistics Canada) [46], limitations due to chronic pain (Statistics Canada) [47], psychological distress (K6) [48], alcohol consumption (Statistics Canada) [46], drug use (Statistics Canada) [46], income (Statistics Canada) [49], perception of financial status (Statistics Canada) [50], met or unmet health needs (new question), feeling of having complicated problems (new question), self-efficacy for managing health (new question). Most questions were initially rated on a 4–5 categorical scale (except for alcohol consumption and drug use).

#### Reference standard: INTERMED self-assessment

IMSA is a self-administered version of the INTERMED questionnaire that helps to measure the complexity of health needs in an adult patient by evaluating the medical, psychological and social spheres. The first version of INTERMED was developed in the 1990s by an international team that combined their research expertise on complexity in order to empirically develop a measuring instrument [51]. Its psychometric qualities [52–54] are well documented. INTERMED presents good validity to predict greater use of services [55–57].

IMSA, which was used in this study, has been available since 2016 [34]. It includes 20 questions subdivided into four domains: Biological, Psychological, Social, and Health system. Every domain is divided into three-time segments: History, Current State, and Vulnerability/Prognosis. Three of the questions have one or more sub-questions. All IMSA items are scored on a four-level rating scale. The rating scores range from 0 to 3, representing no evidence of a symptom, disturbance or healthcare need (0) to evidence of complex symptoms or healthcare needs (3). The maximum total score of the IMSA is 60. A score of 19 or higher indicates complex health needs. A French-language version is available as well as a guide explaining how to complete the questionnaire [58]. The correlations between the total score and the subscales of the IMSA, as compared to the initial INTERMED questionnaire, were high (total score: *r* = 0.79) (95%-CI: [0.70; 0.85]). Cronbach’s α was 0.77, and construct validity was high (SF-36 mental component score: *r* = − 0.57; HADS Depression: *r* = 0.59) [34].

### Analysis

We described continuous variables (age, comorbidity score, IMSA score) using mean +/− standard deviation (SD) and categorical variables (sex, primary language, occupation, marital status, income, and all questions to develop the case-finding tool) using proportions. We tested the 12 selected questions to develop the case-finding tool in bivariate logistic regressions with complexity as measured by the measurement standard (IMSA), as the dependent variable. Significant variables were dichotomized using 2 × 2 tables, based on statistics and team consensus.

These dichotomized variables were then included in a multivariate logistic regression analysis, adjusted for age and sex, and a backward elimination procedure was applied to eliminate those that ceased to be significant in the presence of others. We computed variance inflated factors to check for multicollinearity among the independent variables [59]. We estimated sensitivity and specificity of the different scoring thresholds (number of yes responses) of the case-finding tool when compared to the complex/non-complex classification established by the measurement standard (IMSA). A ROC curve was developed and the area under the curve (AUC) was calculated. We identified the most appropriate threshold score to identify patients with complex health needs [60]. The selected threshold score was the one offering the best compromise between sensitivity and specificity.

To estimate a sensitivity of at least 70% with a 95% confidence level and an accuracy of 10%, 81 complex cases were required (nQuery Advisor® 7.0). Based on previous experience, estimating that the prevalence of patients with complex needs would represent 30% of patients identified, 270 participants had to be recruited. The 189 patients with non-complex needs would provide an accuracy of 6.5% to estimate a specificity of at least 70% [61].

## Results

### Characteristics of the study subjects

**Fig. 1:**
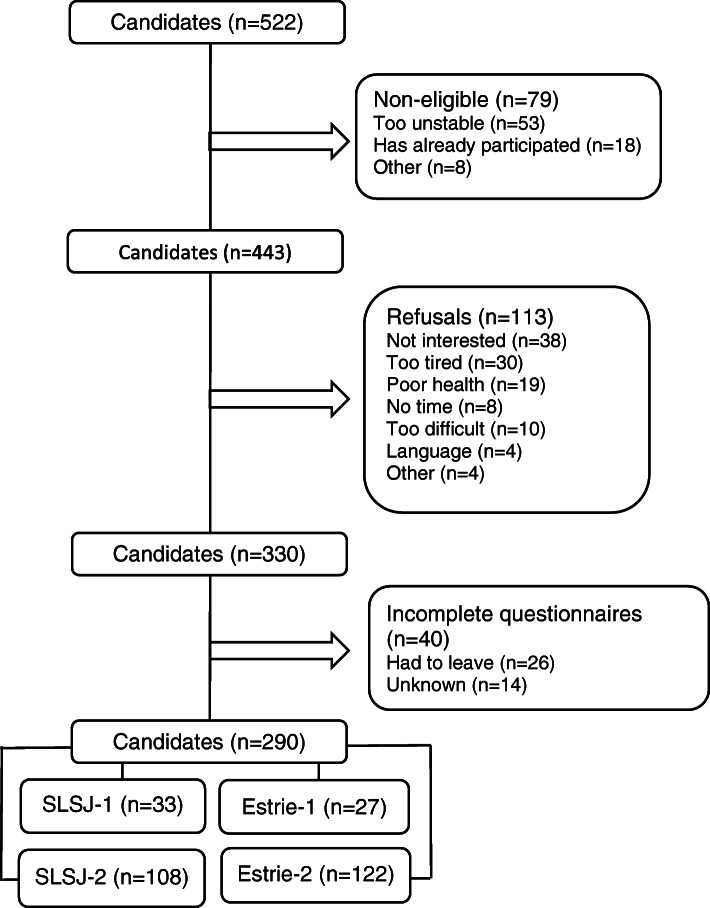
Flow chart of participants

Five hundred twenty-two patients were approached to participate in the study, 79 were not eligible, 113 refused to participate, and 40 had incomplete questionnaires. Two hundred ninety patients participated in the study (see Fig. 1 for flow of participants). Table 1 shows their characteristics. Mean age of participants was 67 (SD = 20.0). Sixty-one percent were female. The primary language of 94.5% of participants was French. Twenty-one percent of the sample were employed. Almost half of the participants were married or living with a partner (45.2%). The average score of the DBMA was 11.8 (SD = 7.2), which corresponded to a high burden [62]. Twenty-six percent of participants (*n* = 75) had complex needs based on the IMSA.

**Table 1:** Patients characteristics

Characteristic	N (%)	Complexity n (%)	
		Not complex *n* = 213 (73%)	Complex *n* = 77 (27%)
Age			
18–34	13 (4)	11 (5)	2 (3)
35–54	49 (17)	36 (17)	13 (17)
55–64	42 (14)	29 (14)	13 (17)
65–74	61 (21)	50 (24)	11 (14)
75–84	83 (29)	58 (27)	25 (32)
85+	42 (15)	29 (13)	13 (17)
Sex			
Female	177 (61)	130 (61)	47 (61)
Male	113 (39)	83 (39)	30 (39)
Primary language			
French	274 (94)	200 (94)	74 (96)
English	13 (5)	11 (5)	2 (3)
Other	3 (1)	2 (1)	1 (1)
Occupation			
Work	61 (21)	56 (26)	5 (6)
Searching for work	2 (1)	1 (1)	1 (1)
School	3 (1)	3 (1)	0 (0)
Do not work for health reasons	44 (15)	19 (9)	25 (32)
At home	10 (3)	9 (4)	1 (1)
Retired	167 (58)	123 (58)	44 (57)
Other	3 (1)	2 (1)	1 (1)
Marital status			
Married or living with a partner	131 (45)	103 (48)	28 (36)
Separated or divorced	38 (13)	26 (12)	12 (16)
Widow	73 (25)	48 (23)	25 (32)
Single	48 (17)	36 (17)	12 (16)
Income			
Less than $20,000	62 (21)	36 (17)	26 (34)
$20,000 - $39,9999	97 (33)	69 (32)	28 (36)
$40,000 - $59,999	68 (23)	55 (26)	13 (17)
$60,000 - $79,999	31 (11)	27 (13)	4 (5)
$80,000 - $99,999	20 (7)	18 (8)	2 (3)
$100,000 and more	9 (3)	6 (3)	3 (4)
Missing	3 (1)	2 (1)	1 (1)

### Main results

**Fig. 2:**
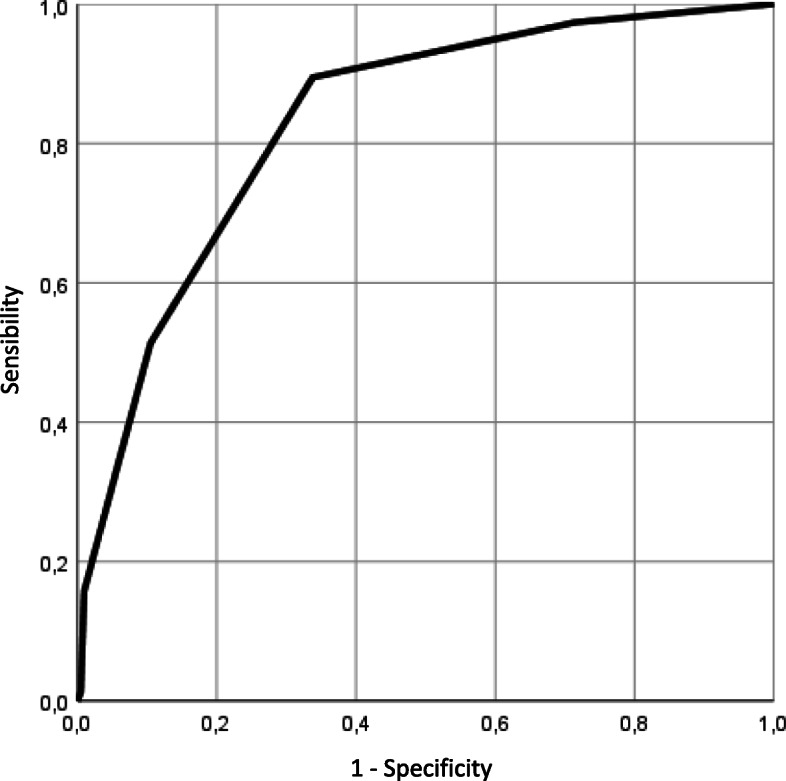
ROC curve with 95% confidence interval

From the initial 12 questions, three were excluded in the bivariate regression, and three others in the multivariate model (see Table 2), yielding a six-question tool, COmplex NEeds Case-finding Tool – 6 (CONECT-6) (see Table 3). All variance inflated factors were lower than 2, showing no sign of multicollinearity. Figure 2 illustrates the ROC curve, with an AUC of 0.84. Using a threshold of two or more positive answers in the six-question tool resulted in a sensitivity and specificity of 90 and 66% respectively. The positive and negative predictive values were 49 and 95% respectively.

**Table 2:** Bivariate and multivariate logistic regressions with complexity as the dependent variable

	Bivariate regression		Multivariate regression	
	Odds ratio	95% Confidence interval	Odds ratio	95% Confidence interval
Perceived healthcare status	9.325	4.652–18.691	7.659	3.429–17.103
Insurance healthcare plan	NS		–	
Social support	2.840	1.235–6.533	4.382	1.460–13.154
Limitations due to chronic pain	3.421	1.661–7.047	2.189	1.137–4.217
Psychological distress (K6)	2.381	1.347–4.208	NS^a^	
Alcohol consumption	NS		–	
Drug use	NS		–	
Income	2.528	1.395–4.580	2.552	1.241–5.248
Perception of financial status	4.293	2.419–7.617	NS^a^	
Met or unmet health needs	3.825	1.450–10.087	2.553	1.261–5.169
Feeling of complicated problems	19.477	4.249–89.280	10.808	2.206–52.956
Self-efficacy for managing health	2.324	1.365–3.957	NS^a^	

*NS* Non significant

^a^Excluded from the mutivariate model because *p* > 0.05 in the presence of the other variables

**Table 3:** The CONECT-6 case-finding tool to identify patients with complex health needs

Questions	Answers	
1. In general, would you say your health is fair or even poor?	Yes	No
2. Do you have pain or discomfort preventing most of your activities?	Yes	No
3. In the past 12 months, do you consider your health needs were met less than half of the time?	Yes	No
4. Do your interactions with the health system and health professionals ever make you feel like you have complex health problems?	Yes	No
5. Is your household income from all sources before taxes and other deductions less than $20,000?	Yes	No
6. In the past 12 months, have you rarely or even never received support from friends or relatives when you needed it?	Yes	No
Number of yes and no answers	**____**	**____**

## Discussion

Case management effectiveness relies on the case-finding process, that is to say the ability of identifying the patients most in need of the intervention [63]. To date, the primary criterion to be recruited in case management programs has mainly relied on a threshold of ED visits. This way of doing presents the advantage of easy measurement, most health centre information systems making this information easily available. However, evidence recommends not only basing the case-finding process on frequent service use, but also on patients’ complex health needs [63]. Indeed, only approximately 35% of frequent users of ED will remain high users in the subsequent year [64, 65]. Moreover, identifying them before their fifth, sixth or more visits to the ED would enable timelier intervention [27].

This study presents the first case-finding tool to identify adult patients with ACSC and complex health needs in EDs. The self-administered format enables the measurement of variables that are not available in electronic medical records or databases, by capturing the patient’s perception (perceived health, social support, met needs, etc.). By using a threshold of 3 ED visits, which is lower than in most reported case management studies [4–11], our case-finding tool helps to identify patients with complex needs upstream and to intervene before they are caught in a vicious cycle of ED frequent use. The sensitivity of these six “yes or no” questions is high (90%), enabling the identification of most frequent users with complex health needs. With a positive predictive value of 49%, approximately one out of two identified patients will obtain a score of complexity on the IMSA. Therefore, a confirmation of complexity is suggested before enrolling them in case management programs. The combination of the case-finding tool and the clinical judgment of healthcare professionals could be a good approach to identify patients for whom case management will likely be most beneficial [63, 66]. Sensitivity, specificity, and positive and negative predictive values are comparable to values obtained for PRISMA-7, a case-finding tool often used to identify older adults with moderate or severe disabilities [31].

### Implications for practice and research

Clinicians and researchers should focus on case-finding processes when implementing or conducting case management [63]. When frequent users of ED with 3 or more visits to the ED are identified as having complex health needs with CONECT-6 (2 yes or more among the 6 questions), they could be referred to a case manager or their primary care team to evaluate the potential benefits of case management if available or other intervention to improve coordination and self-management support [63, 66]. Knowing which questions were answered “yes” could also inform the primary care team on which aspects seem more complex and orient further services. The IMSA could also be used to provide a more accurate picture of complexity in the medical, psychological and social spheres.

## Limitations

Basing this work on the solid expertise of a team conducting research on frequent users, complex needs and case management for many years, we were able to select questions with high potential to build the tool and obtain a 6-question self-administered tool with very good psychometric properties. Four research assistants were involved in data collection and received 3 hours of training for standardization.

This study also has a few limitations. The questions were dichotomized after data collection, and validation was conducted on the same sample than development of the tool. Further validation could be done with this new version of the tool in a different population. We should keep in mind that the tool was validated among patients with ACSC in ED settings. The project was carried out in the winter season, which could have influenced reasons for consultation. But we do not think the seasonal effect had a major impact on results. The study was also carried out in only 2 regions of Quebec. However, the EDs represent a variety of urban sizes, which will help achieve good external validity.

## Conclusions

The case-finding process is the essential characteristic of case management effectiveness. This study presents the first case-finding tool to identify adult patients with ACSC and complex health needs in ED. Other research projects could provide further evidence of the validity of the CONECT-6 tool in contexts other than the ED, and among patients with problems other than ACSC (e.g. mental health problems or other chronic diseases).

## Supplementary Information

**Additional file 1.** The 12 preliminary questions to develop the case-finding tool

## Data Availability

The datasets generated and analyzed during the current study are not publicly available due to the ethics approval but are available from the corresponding author on reasonable request.

## Abbreviations

ACSC: Ambulatory care sensitive condition
AUC: Area under the curve
COPD: Chronic obstructive pulmonary disease
DBMA: Disease Burden Morbidity Assessment
ED: Emergency department
FUED: Frequent user of emergency department
HBP: High blood pressure
IMSA: INTERMED Self-Assessment
ROC: Receiver operating characteristic
