# Correction to: CONECT-6: a case-finding tool to identify patients with complex health needs

**DOI:** 10.1186/s12913-021-06216-7

**Published:** 2021-04-09

**Authors:** Catherine Hudon, Mathieu Bisson, Marie-France Dubois, Yohann Chiu, Maud-Christine Chouinard, Nicole Dubuc, Nicolas Elazhary, Véronique Sabourin, Alain Vanasse

**Affiliations:** 1 Department of Family Medicine and Emergency Medicine, University of Sherbrooke, 3001 12e Avenue N, Sherbrooke, QC J1H 5H3 Canada; 2 Nursing Faculty, University of Montreal, Pavillon Marguerite-d’Youville, C.P. 6128 succ. Centre-ville, Montréal, QC H3C 3J7 Canada; 3 Integrated University Health and Social Services Centre of Saguenay–Lac-Saint-Jean, 930 rue Jacques-Cartier E, Chicoutimi, QC G7H 7K9 Canada

**Correction to: BMC Health Serv Res 21, 157 (2021)**

**https://doi.org/10.1186/s12913-021-06154-4**

Following publication of the original article [1], the authors would like to make some changes.

1. The percentage of participants in the Results under the Abstract section needs to be corrected.

The sentence currently reads:

The positive and negative predictive values were 49 and 75% respectively.

The sentence should read:

The positive and negative predictive values were 49 and 95% respectively.

2. The AUC has disappeared from Figure 2 (ROC curve) due to a typesetting mistake. The correct Figure 2 is shown below:

**Fig. 2:**
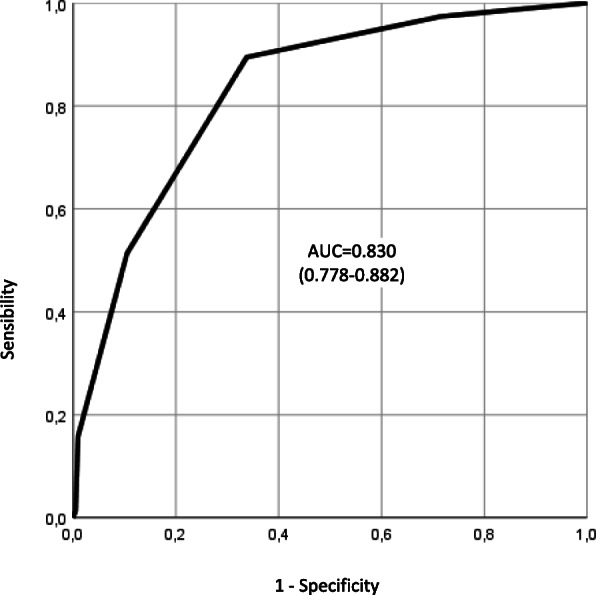
ROC curve with 95 pct confidence interval

3. There are currently two affiliations associated to Catherine Hudon, which are actually a duplicate (with a minor difference in the address). The author affiliation 2 needs to be removed, so that the correct authors affiliations are presented as below:

Catherine Hudon1*, Mathieu Bisson1, Marie-France Dubois1, Yohann Chiu1, Maud-Christine Chouinard2, Nicole Dubuc1, Nicolas Elazhary1, Véronique Sabourin3, Alain Vanasse1

1. Department of Family Medicine and Emergency Medicine, University of Sherbrooke, 3001 12e Avenue N, Sherbrooke, QC, Canada, J1H 5H3.

2. Nursing Faculty, University of Montreal, Pavillon Marguerite-d'Youville, C.P. 6128 succ. Centre-ville, Montréal, QC, Canada, H3C 3J7.

3. Integrated University Health and Social Services Centre of Saguenay–Lac-Saint-Jean, 225 rue Saint-Vallier, Chicoutimi, Quebec, G7H 5H6

The original article has been corrected.

## References

[1] Hudon C (2021). CONECT-6: a case-finding tool to identify patients with complex health needs. BMC Health Serv Res.

