# Supplementary material for: CONECT-6: a case-finding tool to identify patients with complex health needs
Source: BMC Health Serv Res. 2021 Feb 17;21:157. doi: 10.1186/s12913-021-06154-4 (PMC7891167; doi:10.1186/s12913-021-06154-4)
Supplement: Supplementary file 1 — Additional file 1. The 12 preliminary questions to develop the case-finding tool [file 12913_2021_6154_MOESM1_ESM.docx]

**Additional file 1. The 12 preliminary questions to develop the case-finding tool**

1. **In general, would you say your health is...?**

 Excellent

 Very good

 Good

 Fair

 Poor

1. **Which insurance program covering all or part of the cost of your prescription medications describes your situation?**

 Government's prescription medications insurance program (RPAM/RAMQ) for recipient of last resort assistance (welfare)

 Government's prescription medications insurance program (RPAM/RAMQ) for people aged 65 or over

 Government's prescription medications insurance program (RPAM/RAMQ) for people aged less than 65

 Private program (employer, collective, other than government)

**In the past 12 months, how often did you receive support from friends or relatives when you needed it?**

 Almost always

 Frequently

 Half the time

 Rarely

 Never

1. **What is your level of activity limitation due to pain or discomfort?**

 No pain or discomfort

 Pain that does not prevent any activity

 Pain preventing few activities

 Pain preventing some activities

 Pain preventing most of activities

|  | **In the past 30 days, how often did you feel…** | All the time | Most of the time | Some-times | Rarely | Never |
| --- | --- | --- | --- | --- | --- | --- |
|  | … nervous? |  |  |  |  |  |
|  | … desperate? |  |  |  |  |  |
|  | … agitated? |  |  |  |  |  |
|  | … depressed? |  |  |  |  |  |
|  | … exhausted? |  |  |  |  |  |
|  | … good-for-nothing? |  |  |  |  |  |

1. **In the past 12 months, how often did you drink alcoholic beverages?**

 Less than once a month

 Once a month

 2-3 times a month

 Once a week

 2-3 times a week

 4-6 times a week

 Everyday

|  | **In the past 12 months, how often did you use…** | Not once | 1-3 times | Once a week | More than once a week | Everyday |
| --- | --- | --- | --- | --- | --- | --- |
|  | … marijuana, cannabis or hashish? |  |  |  |  |  |
|  | … cocaine or crack? |  |  |  |  |  |
|  | … speed (amphetamines)? |  |  |  |  |  |
|  | … ecstasy (MDMA)? |  |  |  |  |  |
|  | … hallucinogenic drugs, PCP or LSD (acid)? |  |  |  |  |  |
|  | … glue, gasoline or other solvents? |  |  |  |  |  |
|  | … heroin? |  |  |  |  |  |
|  | … steroids? |  |  |  |  |  |

1. **In total, your household income from all sources before taxes and other deductions is:**

 Less than $20,000

 $20,000 to $39,999

 $40,000 to $59,999

 $60,000 to $79,999

 $80,000 to $99,999

 $100,000 and more

1. **How do you perceive your financial situation compared to people of your age?**

 I consider myself financially **comfortable**.

 I consider my income **sufficient** to meet my needs and/or those of my family (housing, food, medicine, etc.).

 I consider my income **insufficient** to meet my needs and/or those of my family (housing, food, medicine, etc.).

 I consider my income **very inadequate** to meet my needs and/or those of my family (housing, food, medicine, etc.).

1. **In the past 12 months, how often do you consider that your health needs were met?**

 Almost always

 Frequently

 Half the time

 Rarely

 Never

**How often do your interactions with the health system and healthcare professionals make you feel that you have complex health problems?**

 Never

 Rarely

 Half the time

 Frequently

 Almost always

1. **How confident are you of being able to do the different tasks and activities needed to manage your health?**

 Totally confident

 Very confident

 Rather confident

 Not very confident

 Not at all confident
